# Chitosan-coated silver nanoparticles amplify glycopeptide efficacy against *Staphylococcus aureus*

**DOI:** 10.3389/fmicb.2026.1787175

**Published:** 2026-06-24

**Authors:** Sakshi V. Khairnar, Anna Sommers, Elena Muldiiarova, Kenneth W. Bayles, David Oupický, Vinai C. Thomas, Marat R. Sadykov, Svetlana Romanova

**Affiliations:** 1 Center for Drug Delivery and Nanomedicine, Department of Pharmaceutical Sciences, University of Nebraska Medical Center, Omaha, NE, United States; 2 Department of Pathology, Microbiology and Immunology, University of Nebraska Medical Center, Omaha, NE, United States

**Keywords:** antibiotic resistance, antimicrobial agents, glycopeptide antibiotics, silver nanoparticles, *Staphylococcus aureus*, vancomycin

## Abstract

The escalating prevalence of antibiotic resistance represents a critical threat to global public health, while the development of new antibiotics remains limited. Vancomycin, a glycopeptide antibiotic, is essential for treating severe Gram-positive infections. However, the emergence of vancomycin-intermediate (VISA) and vancomycin-resistant (VRSA) *Staphylococcus aureus* strains has raised significant concerns due to their challenging resistance profiles and limited treatment options. In this study, we developed a carboxylic acid-capped silver nanoparticle (AgNPs) platform to enhance the therapeutic delivery and efficacy of vancomycin. The nanocomposite (AgNPs/Van/CS) was engineered by leveraging electrostatic interactions between AgNPs and vancomycin, with a chitosan coating to provide stability, bacterial adhesion, and improved biocompatibility. Our results demonstrate that AgNPs/Van/CS significantly outperformed vancomycin alone, exhibiting enhanced antibacterial and antibiofilm activity against both methicillin-sensitive (MSSA) and methicillin-resistant (MRSA) *S. aureus* strains, as well as vancomycin-intermediate (VISA) clinical isolates. Notably, AgNPs/Van/CS achieved potent bacterial inhibition at lower antibiotic concentrations and significantly reduced biofilm formation, while maintaining strong activity under diverse laboratory conditions. These findings highlight the potential of AgNPs/Van/CS as a promising strategy for managing *S. aureus* infections and addressing the urgent challenge of antibiotic resistance.

## Graphical Abstract

**Figure:**
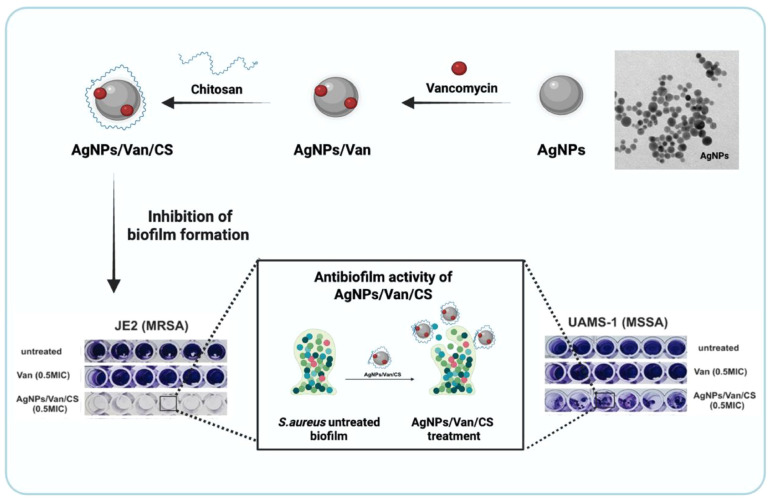
Illustrating AgNPs functionalization with vancomycin and chitosan, resulting in enhanced antibiofilm activity against MRSA and MSSA.

## Introduction

Bacterial pathogens continue to pose a significant global health challenge, causing a broad spectrum of illnesses ranging from mild respiratory infections to life-threatening sepsis. This burden results in substantial morbidity across all age groups, increased hospitalization rates and disability-adjusted life years (DALYs) (Antimicrobial Resistance Collaborators, 2022; Gasser et al., 2023). The therapeutic landscape for bacterial infections has become increasingly complex due to the alarming rise of antimicrobial resistance (AMR). According to projections by the Organization for Economic Cooperation and Development (OECD), the prevalence of resistance to antibiotics of last resort is expected to double between 2005 and 2035 (OECD, 2025). In 2019, an estimated 4.95 million deaths were associated with bacterial AMR, including 1.27 million deaths directly attributable to such resistance (Antimicrobial Resistance Collaborators, 2022). The economic impact is also severe, with high-income countries facing average costs exceeding US$ 32,000 per sepsis patient (World Health Organization, 2025d), and healthcare costs attributable to AMR projected to reach up to US$ 1 trillion annually by 2050 (World Health Organization, 2025b).

Among the multitude of bacterial pathogens, methicillin-resistant *Staphylococcus aureus* (MRSA) stands out as a major driver of the global AMR crisis (Abebe and Birhanu, 2023). *S. aureus* is responsible for a wide range of infections, from superficial skin infections to severe, invasive diseases such as pneumonia, endocarditis, and sepsis (Tong et al., 2015). It’s remarkable ability to rapidly develop resistance to multiple antibiotics has made it a formidable public health threat (Gherardi, 2023; Rungelrath and DeLeo, 2021). *S. aureus* has evolved diverse mechanisms to evade antibiotic action, including enzymatic modification or degradation of antibiotics, efflux pumps that actively expel antibiotics from bacterial cells, alterations in cell envelope permeability, modifications of antibiotic target sites, and the use of alternative metabolic pathways to bypass antibiotic effects (Brdová et al., 2024; Chambers and DeLeo, 2009; Peterson and Kaur, 2018). The escalation of multidrug-resistant (MDR) pathogens is epitomized by MRSA, which is defined by its resistance to nearly all β-lactam antibiotics and is frequently resistant to multiple other antimicrobial classes (Lakhundi and Zhang, 2018; Magiorakos et al., 2012). Recent global surveillance data reveal the extent of this crisis: in 2019, MRSA alone was responsible for over 100,000 deaths and 3.5 million DALYs worldwide, making it the leading pathogen-drug combination causing deaths attributable to AMR (Antimicrobial Resistance Collaborators, 2022).

Vancomycin, a glycopeptide antibiotic, remains the first-line therapy for MRSA infections, particularly for bacteremia and endocarditis (Choo and Chambers, 2016). However, MRSA strains have shown increasing resistance to vancomycin, raising concern given vancomycin’s central role in both community and health care-related settings (Hidayat et al., 2006). Vancomycin exerts its antibacterial activity by inhibiting bacterial cell wall synthesis, binding to the D-alanyl-D-alanine (D-Ala-D-Ala) portion of peptidoglycan precursors, and preventing cross-linking of peptidoglycan chains (Patel et al., 2024). The prevalence of vancomycin-resistant *S. aureus* (VRSA), vancomycin-intermediate *S. aureus* (VISA), and heterogeneous VISA (hVISA) has been increasing globally, with the first case of VRSA in the United States reported in 2002 (Centers for Disease Control and Prevention [CDC], 2002; Shariati et al., 2020). Although the cases remain rare, laboratory evidence suggests that resistance could spread if selective pressures persist (Howden et al., 2010).

Various promising strategies are being investigated to address the challenge of AMR in *S. aureus*, including the discovery of novel antibiotics, enhancement of the efficacy of existing therapies, and the development of alternatives such as bacteriophage, antibody therapies, anti-biofilm drugs, nanomaterials, and vaccines (Ye and Chen, 2022). Despite the efforts, the pipeline for new antibiotics remains alarmingly thin. As of 2021, only 27 antibiotics were in clinical development against priority pathogens, with only six fulfilling at least one of the World Health Organization’s criteria for innovation (World Health Organization, 2025a,c).

Developing more efficient delivery systems can further enhance the efficacy of antibiotics against *S. aureus*. This might include using nanoparticles or other advanced drug delivery technologies to enhance antibiotic penetration and targeting (Mondal et al., 2024). Metal and metal oxide nanoparticles, especially silver, gold, zinc oxide, and copper oxide nanoparticles, exhibit potent antibacterial properties through mechanisms such as membrane damage and reactive oxygen species (ROS) generation, which can help overcome existing resistance mechanisms (Khairnar et al., 2025; Mba and Nweze, 2021). Silver nanoparticles (AgNPs), in particular, have demonstrated efficient antibacterial activity against both gram-negative and gram-positive bacteria, including *S. aureus* (Rai et al., 2009). However, concerns about toxicity and environmental impact have limited their widespread clinical use (Marambio-Jones and Hoek, 2010).

To address these challenges, our study utilized a carboxylic acid-capped AgNPs platform for therapeutic delivery. This system was engineered to enhance the effectiveness of antimicrobial agents by leveraging the electrostatic interaction between AgNPs and vancomycin, with chitosan coatings providing stability and improved biocompatibility (Ahmed et al., 2016; Aktug et al., 2021; Hajipour et al., 2012; Yan et al., 2021). The resulting AgNPs/Van/CS nanocomposite demonstrated significantly enhanced antibacterial and antibiofilm efficacy against both methicillin-sensitive and methicillin-resistant *S. aureus* strains compared to vancomycin alone, achieving potent inhibition at lower antibiotic concentrations. Notably, it was also effective against MSSA, MRSA, and VISA clinical isolates, highlighting AgNPs/Van/CS as a promising nanoplatform for improving vancomycin efficacy, reducing required antibiotic doses, and providing a potent strategy for combating challenging *S. aureus* infections and mitigating antibiotic resistance.

## Experimental

### Materials

Silver nitrate was purchased from Fisher Scientific (Waltham, MA, USA). Succinic acid anhydride and chitosan were obtained from Sigma-Aldrich (St. Louis, MO, USA). Dansyl chloride was purchased from Fluka (Buchs, Switzerland), and MTT [3-(4,5-dimethylthiazol-2-yl)-2,5-diphenyltetrazolium bromide] was obtained from Combi-Blocks (San Diego, CA, USA). Double-distilled water was used throughout all experiments. All other chemicals and reagents were of analytical grade or equivalent quality. Fetal bovine serum (FBS), DMEM High Glucose medium, and penicillin/streptomycin were purchased from Invitrogen (Carlsbad, CA, USA).

### Preparation and characterization of silver-based nanoparticles

#### Synthesis and characterization of silver nanoparticles (AgNPs)

Carboxylic acid-capped AgNPs were synthesized employing a chemical reduction method adapted from a recent report (Irfan et al., 2022). Briefly, 30 mL 1 mM succinic acid was used as a capping agent in the presence of 60 mL 1 mM NaBH_4_ as the reducing agent for the 20 mL 1 mM AgNO_3_ precursor. The synthesized AgNPs were purified by centrifugation at 10,000 rpm, and their formation was confirmed using UV-Vis spectroscopy and dynamic light scattering (DLS).

#### Preparation of complexes (AgNPs/Van) and coated complex (AgNPs/Van/CS)

Complexes composed of AgNPs and vancomycin (AgNPs/Van) were prepared by mixing an aqueous solution of AgNPs at a constant Ag concentration (5.78 ± 1.14 μg/mL) with vancomycin from 0.05 to 1.0 mM concentrations, resulting in different compositions. The complexation process was carried out over 24 h at room temperature (RT) with gentle agitation to ensure optimal interaction between the nanoparticles and the antibiotic. The progress of the complexation reaction was followed using UV-Vis spectroscopy to identify the time and ratio at which the reaction reached complete complexation. For coating, chitosan solution was prepared in 1% acetic acid at 0.2% w/v (Farhadi, 2022). Insoluble particles were removed using a 0.22 μm filter. The complex solution was slowly added to the chitosan stock solution with continuous stirring for 24 h before purification by centrifugation to remove uncoated materials.

### Characterization of nanoparticles

#### UV-Vis spectra

UV-Vis absorption spectra were recorded using a SpectraMax iD5 Multi-Mode Microplate Reader (Molecular Devices, San Jose, CA, USA). Samples were loaded into 1 mL quartz cuvettes with a 1 cm path length. Spectra were collected over the wavelength range of 250–600 nm. Measurements were performed at RT, using double-distilled water as the blank. Spectra were recorded with a data interval of 1 nm. All measurements were conducted in triplicate, and baseline correction was applied.

#### DLS and zeta-potential

Effective hydrodynamic diameters (D_*eff*_), polydispersity indexes (PDI), and ζ-potential of the particles were determined by DLS using Zetasizer Nano ZS (Malvern Instruments Ltd., Malvern, UK). All measurements were performed in triplicate at 25 °C with 10 scans and 120 s equilibration time.

#### Nanoparticle tracking analysis (NTA)

Nanoparticle tracking analysis measurements were performed with Nanosight NS300 (Malvern, UK). All samples were diluted in PBS to a final volume of 1 mL. Ideal measurement concentrations were found by pre-testing the particle per frame value (20–100 particles/frame). The camera level was increased until all particles were distinctly visible (camera level 14). For each measurement, five 1-min videos were captured under the following conditions: cell temperature: 25 °C; syringe speed: 20 μL/s. After capture, the videos were analyzed using the build-in Nano Sight Software NTA 3.1. The detection threshold was determined as 4. Measurements were conducted in light scatter mode using the built-in 488 nm laser module.

#### Transmission electron microscopy (TEM)

Samples for TEM imaging were spotted onto formvar/silicon monoxide-coated 200 mesh copper grids (Ted Pella Inc., Redding, CA). Grids were glow-discharged for 1 min at 20 μA with a GloQube glow discharge unit (Quorum Technologies, East Sussex, UK) prior to use. Samples were negatively stained with NanoVan (Nanoprobes, New York, NY) and examined on a Tecnai G2 Spirit TWIN (FEI, Hillsboro, OR) operating at an accelerating voltage of 80 kV. Images were acquired digitally with an AMT (Woburn, MA) digital imaging system.

#### Fourier transform infrared spectroscopy (FT-IR)

Fourier transform infrared spectroscopy spectrometer (Nicolet iS 5 with an iD7 attenuated total reflectance accessory, Thermo Fisher Scientific Inc., MA, USA) was used to characterize the samples. The operating spectral range was 400–4000 cm^–1^.

### Quantitative concentration determination of silver and vancomycin

#### Inductively coupled plasma mass spectrometry (ICP-MS)

Silver content determination was performed on ICP-MS (NexION 300Q, PerkinElmer, USA) instrument. Concentrated Nitric acid (HNO_3_, 70%, Optima Grade) was used for sample digestion, and ultra-pure deionized water with a resistivity of 18.2 MΩ⋅cm at 25°C was used for sample preparation and dilutions. The Ag standard solutions required in the experiment were prepared by stepwise dilution. The NexION setup solution containing Be, Ce, Fe, In, Li, Mg, Pb, and U (1 μg/L) in 1% HNO 3 for ICP-MS condition optimization was purchased from PerkinElmer (USA). The purity of argon used in ICP-MS is higher than 99.999%.

For ICP-MS measurements, the instrument was set with a radiofrequency power of 1150 W, a nebulizer gas flow of 0.92 L/min, and an auxiliary gas flow of 1.2 L/min. Argon was used as the carrier gas. The cooling gas flow was maintained at 18 L/min, with the plasma gas flow also set at 18 L/min and an RF power of 1600 W. The sample flow rate was adjusted to 40 μL/min. Data acquisition was for 100 s, with a dwell time of 100 μs and 5 ms depending on the measurement cycle. The detector was operated in dual mode. Silver (^107^Ag isotope) was determined in standard measurement mode with three replicates.

#### Ultra-performance liquid chromatography (UPLC)

The vancomycin content in final formulations was determined using ACQUITY H-Plus UPLC system (Waters, Milford, MA) equipped with a photodiode array (PDA) detector set at 280 nm. As stationary phase, an ACQUITY UPLC BEH C18 1.7 μm column was used (2.1 mm × 50 mm). The chromatographic separation was performed using a binary solvent system adapted from Shou et al. (2014). The mobile phase was maintained at a flow rate of 0.3 mL/min, containing 0.1% (v/v) formic acid water solution (A) and 0.1% (v/v) formic acid acetonitrile solution (B). The gradient began with 95.0% eluent A for 1 min, changed to 60.0% for 3 min, changed to 1% for 3 min, and changed back to 95% for 3 min. The separations were completed within 10 min. The temperature of the column was maintained at 45 °C. Vancomycin concentration was calculated via a standard calibration curve of vancomycin ranging from 6.25 to 200 μg/mL.

### Quantification of surface carboxylic groups

Fluorescent labeling technique was employed for succinic acid quantification (Bartzatt, 2001). Briefly, 150 μL of each sample was transferred into individual wells of a 96-well microplate, followed by the addition of 100 μL of dansyl chloride solution (5 mg/mL in acetone). The plate was incubated at 20 °C in the dark for 1 h to facilitate the dansylation of surface carboxylic acid groups. After the reaction, fluorescence was measured using a microplate reader with an excitation wavelength of 337 nm and an emission wavelength of 535 nm.

### Stability studies

The stability of the AgNPs formulations was assessed by monitoring particle size and PDI using DLS. For AgNPs, measurements were performed daily for 60 days while samples were stored at 4 °C. For AgNPs/van and AgNPs/van/CS, particle size was recorded over a 14-days period under identical storage conditions. To evaluate pH stability, the pH of AgNPs was adjusted to 3.0, 5.0, 7.0, 9.0, and 11.0 using either HCl or NaOH. Following pH adjustment, particle size was measured immediately, and then again at 24 h, 48 h, and 72 h post-adjustment.

### *In vitro* cytotoxicity

The cytotoxicity of formulations was evaluated using the MTT assay. The assay was conducted on the L-929 murine fibroblast cell line, kindly provided by the Bin Duan laboratory, University of Nebraska Medical Center. The cell lines were cultured in a high-glucose DMEM medium containing 10% FBS, penicillin (100 U/mL), and streptomycin (100 μg/mL) at 37 °C with 5% CO2 in a humidified chamber. Cells were seeded at a density of 5,000 cells per well in a 96-well plate and allowed to adhere overnight. The following day, cells were treated with varying concentrations of the formulations, corresponding to vancomycin equivalent concentrations ranging from 0.5 to 64 μg/mL. After 24 h of incubation, MTT reagent was added, and the cells were further incubated to allow for formazan crystal formation. Absorbance was measured at 570 nm using a microplate reader to determine cell viability relative to untreated controls.

### Hemolytic activity

The blood samples were collected from C57BL/6 mice in tubes containing heparin as an anticoagulant. This procedure was performed in accordance with approved protocol 24-093-02-FC by the Institutional Animal Care and Use Committee at the University of Nebraska Medical Center. Pooled blood was centrifuged at 1,500 × *g* for 5 min, and the plasma fraction was carefully removed. The resulting pellets were washed twice by resuspending in an equal volume of saline and mixing by inversion, followed by centrifugation at 1,500 × *g* for 5 min. After the final wash, the red blood cells (RBCs) were resuspended in PBS and diluted to obtain 1% (v/v) suspension. The assay was conducted in a 96-well microplate following previously published procedures (Sæbø et al., 2023). PBS and 1% Triton X-100 served as negative (diluent) and positive (complete hemolysis) controls, respectively. For each well, 100 μL of 1% RBC suspension and 100 μL of test compound were used. Samples were incubated on a shaker for 1 h (37 °C, 100 rpm). Measurements were taken in triplicate. After incubation, samples were centrifuged at 1,500 × *g* for 5 min at room temperature, and 150 μL of the supernatants were transferred to a flat-bottom 96-well plate. Absorbance was measured at 405 nm using a multi-mode plate reader. Hemolysis was assessed by measuring the percentage of released hemoglobin (%). Samples were classified as non-hemolytic (0%–2%), slightly hemolytic (2%–5%), or hemolytic (>5%) (van Oeveren, 2013).

Percentage hemolysis was calculated using the following equation:


%haemolysis=

$$\frac{\left(\mathrm{absorbance}\,\mathrm{of}\,\mathrm{test}\,\mathrm{sample}\right)-\left(\mathrm{absorbance}\,\mathrm{of}\,\mathrm{diluent}\right)}{\left(\mathrm{absorbance}\,\mathrm{of}\,\mathrm{positive}\,\mathrm{control}\right)-\left(\mathrm{absorbance}\,\mathrm{of}\,\mathrm{diluent}\right)}\times 100.$$

### Bacterial strains and growth conditions

The strains used in this study were obtained from our laboratory collection and included methicillin-resistant *S. aureus* (MRSA) strains USA300 LAC JE2 and methicillin-susceptible *S. aureus* (MSSA) strain UAMS-1. Vancomycin-intermediate *S. aureus* (VISA), MRSA, and MSSA clinical isolates were obtained from the Clinical Microbiology Laboratory at Nebraska Medicine. Bacterial cultures were grown in tryptic soy broth (TSB) medium supplemented with 0.25% glucose or on TSB plates containing 1.5% agar. MIC values for AgNPs/Van and AgNPs/Van/CS were expressed as vancomycin-equivalent concentrations (μg/mL). The vancomycin content associated with the nanoparticle formulations was quantified by UPLC method presented in Table 1. The vancomycin MIC breakpoints for *S. aureus* were applied according to current clinical standards as follows: susceptible (VSSA), MIC ≤ 2 μg/mL; intermediate (VISA), MIC 4–8 μg/mL; and resistant (VRSA), MIC ≥ 16 μg/mL. Accordingly, for the *S. aureus* strains used in this study, the MIC values were set at 2 μg/mL for MSSA and MRSA and 4 μg/mL for VISA (Holmes et al., 2012; Tenover and Moellering, 2007). *S. aureus* cultures were incubated overnight in 3 mL of TSB at 37 °C with shaking at 250 rpm. The overnight cultures were then diluted in fresh TSB medium to an optical density at 600 nm (OD_600_) of 0.05 (∼4 × 10^7^ CFU/mL). Growth analysis was performed in a 96-well plate using an Infinite 200 Pro Tecan spectrofluorometric plate reader at 37 °C with the maximum aeration setting. Optical density at 600 nm was recorded every 30 min for 24 h. Measurements were performed in quadruplicate, with 200 μL of culture per well and at least three technical replicates per run. For anaerobic growth assays, 50 μL of sterile mineral oil was directly layered on to cultures in a 96-well plate and growth was monitored every 30 min without agitation.

**TABLE 1:** Quantitative determination of silver and vancomycin content.

Sample	*^a^*[Ag], μ g/mL	*^b^*C(Vanc), μ g/mL
AgNPs	5.78 ± 1.14	–
AgNPs/Van	8.83 ± 0.25	142.83 ± 5.25
AgNPs/Van/CS	6.19 ± 0.65	127.37 ± 5.05

*^a^*Determined by ICP-MS.

*^b^*Determined by UPLC.

### Time-kill assay

Exponential phase cultures of *S. aureus* JE2 and UAMS-1 were inoculated into TSB at a final inoculum of 5 × 10^5^ CFU/mL and subjected to various concentrations of vancomycin or AgNPs/Van/CS (2×, 1×, 0.75×, 0.5×, and 0.25× vancomycin MIC corresponding to 4, 2, 1.5, 1, and 0.5 μg/mL of vancomycin). Cultures were incubated at 37 °C and 250 rpm for 24 h. An untreated control was included for each strain. At 1, 3, 6, and 24 h post-treatment, aliquots were collected, diluted 1:2 in sterile charcoal (25 mg/mL), and incubated on ice for 15 min to minimize drug carryover (Belley et al., 2008). Samples were then serially diluted and plated on TSA to determine CFU/mL. Data represents three biological replicates and were analyzed using GraphPad Prism.

### Static biofilm assay

Static biofilms were grown as previously described (Majerczyk et al., 2008). Sterile polystyrene 96-well flat-bottom microtiter plates (Corning, Inc.) were pre-coated with 100 μL of 20% human plasma in carbonate buffer and incubated overnight at 4°C. Bacterial cultures were then diluted in fresh TSB supplemented with 0.5% glucose and 3% sodium chloride to an OD_600_ of 0.05. A total of 200 μL of the diluted cultures was used to inoculate the wells of the pre-coated microtiter plates, and biofilms were grown statically for 24 h at 37°C. After incubation, the medium and nonadherent bacteria were removed, and the wells were washed twice with PBS. Adherent bacteria were fixed in the microtiter plate with 100 μL of 100% ethanol for 2 min, followed by staining with 100 μL of crystal violet (CV) solution for 2 min at room temperature. The wells were then washed three times with PBS. The quantification of biofilm biomass stained by CV was performed using an Infinite 200 Pro Tecan spectrofluorometric plate reader at an absorbance of 595 nm (A_595_).

### Statistical data analysis

Statistical comparisons were carried out using One-Way ANOVA with multiple comparison test using GraphPad Prism 6 significant (GraphPad Software, Inc., San Diego, CA). *P*-values less than 0.05 were considered.

## Results and discussion

### Preparation and characterization of AgNPs/Van/CS

**SCHEME 1:**
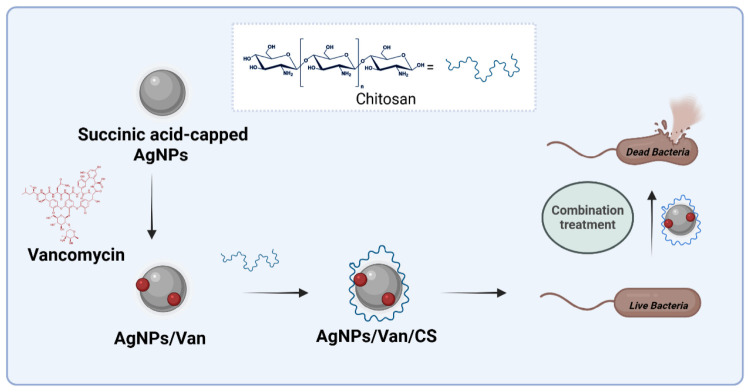
Schematic representation of the preparation of formulation utilizing silver nanoparticles (AgNPs) as a carrier for vancomycin to treat multi-drug resistant bacteria.

In this study, AgNPs were successfully prepared and utilized to develop antibacterial formulations, as illustrated in Scheme 1. The process began with the reduction of AgNO_3_ using NaBH_4_ in the presence of succinic acid, which served as both a stabilizing agent and a source of carboxyl groups for further functionalization. The successful reduction of Ag^+^ to Ag^0^ was indicated by a color change from colorless to light yellow. The carboxyl-capped AgNPs can be readily subjected to electrostatic interaction with the amine group of vancomycin for secondary functionalization. This complex was further enhanced by coating with chitosan, leveraging the electrostatic attraction between the positively charged chitosan and the negatively charged surface.

**FIGURE 1:**
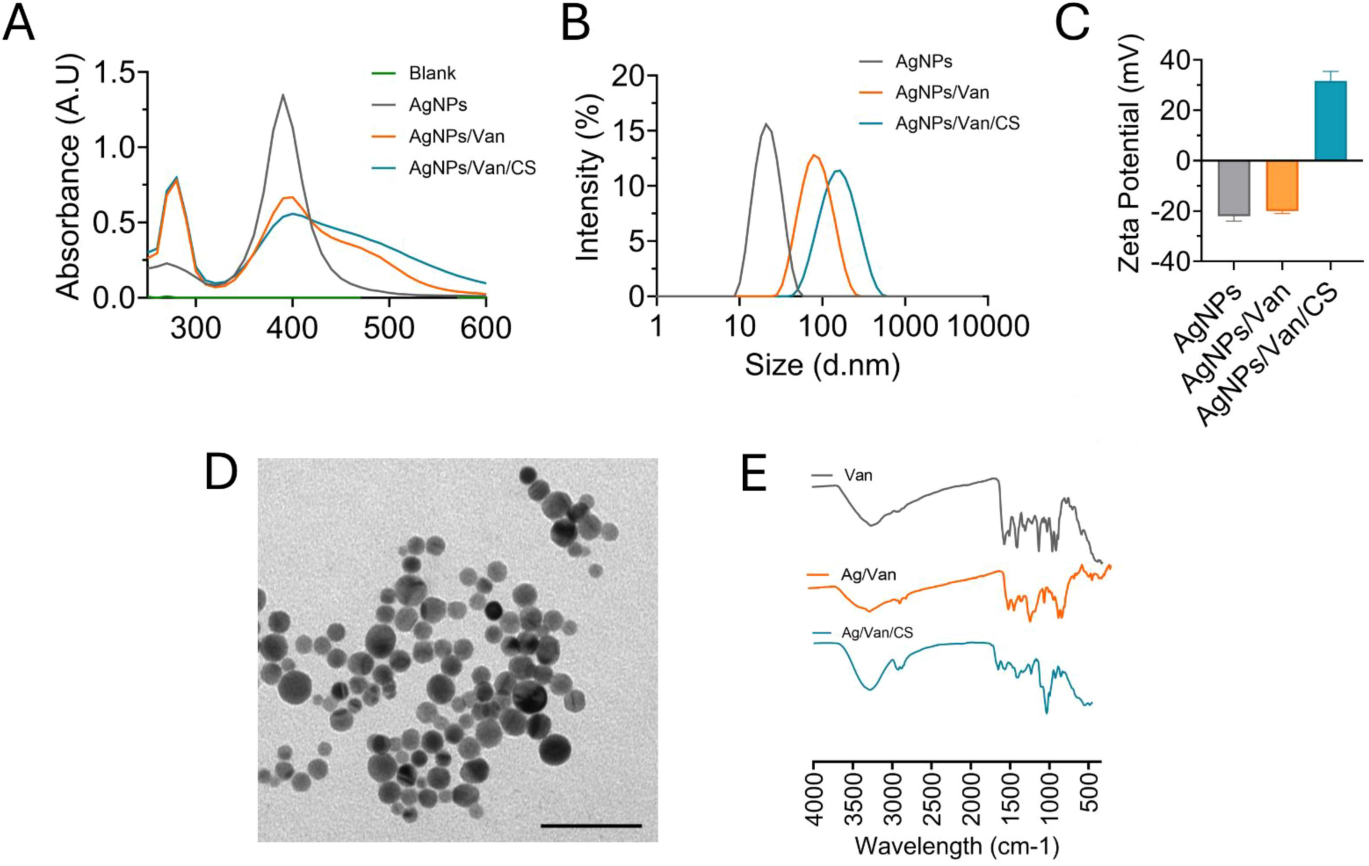
Characterization of AgNPs and functionalized nanocomposites. **(A)** UV-Vis spectra, revealing the characteristic surface plasmon resonance peak of AgNPs and the absorption band of vancomycin. **(B)** Dynamic light scattering (DLS) analysis depicting the hydrodynamic size distribution of bare AgNPs, vancomycin-functionalized AgNPs, and chitosan-coated complexes. **(C)** Zeta potential measurements illustrate the surface charge modifications across different stages of nanoparticle functionalization. **(D)** Transmission electron microscopy (TEM) image of AgNPs at RT, Scale bar: 50 nm. **(E)** Fourier transform infrared (FTIR) spectra confirming the successful functionalization and surface interactions. All measurements were performed in triplicate, and data are presented as mean ± standard deviation unless otherwise noted.

The UV-Vis spectra (Figure 1A) were consistent with surface modification. The AgNPs exhibited a characteristic surface plasmon resonance (SPR) band at 398 nm. Upon vancomycin complexation, vancomycin’s absorption band was detected at 280 nm, accompanied by a 5–10 nm bathochromic shift in the AgNPs SPR band. This shift is attributed to alterations in surface chemistry and electronic transitions (Kaur et al., 2018). The average hydrodynamic diameter measured by DLS is shown in Figure 1B, where the bare AgNPs exhibited a size of 20 ± 1 nm and a PDI of 0.2. Functionalization with vancomycin increased the size to 85 ± 10 nm, while subsequent chitosan coating further enlarged the particles to 120 ± 5 nm. Zeta potential measurements (Figure 1C) provided insights into surface charge modifications. Bare AgNPs and AgNPs/Van complexes showed negative zeta potentials (−25 ± 5 mV and −20 ± 2 mV, respectively) due to the presence of carboxyl groups. Chitosan coating shifted the zeta potential to a positive value (30 ± 5 mV), demonstrating successful surface modification. TEM imaging (Figure 1D) revealed spherical AgNPs with an average diameter of 20 ± 2 nm. NTA measurements revealed 32 ± 1 nm as the mean size of AgNPs with a particle concentration of 2.03e+08 ± 8.31e+07 particles/mL. FT-IR spectra of vancomycin, AgNPs/Van and AgNPs/Van/CS (Figure 1E) confirmed effective interaction between vancomycin and AgNPs. Spectrum of vancomycin hydrochloride showed characteristic peaks at 3406 cm^–1^ of the hydroxyl stretching, 1658 cm^–1^ of the *C* = O stretching, 1502 cm^–1^ of the *C* = C, and 1230 cm^–1^ of the phenolic hydroxyl groups (Mohamed et al., 2017). In formulation, the peaks at 605 cm^–1^ (Ag-O stretch) and 1550–1650 cm^–1^ (CS N–H bend) were also observed (Begum et al., 2022).

### Determination of vancomycin and Ag content in AgNPs/Van/CS

The combination of advanced analytical techniques was employed to quantify the each components of the formulation. These methods were selected for their high sensitivity, specificity, and ability to detect trace amounts in complex biological samples. For silver content analysis, ICP-MS was utilized. This technique offers exceptional sensitivity for detecting metals and other elements, such as phosphorus or sulfur (Fuentes-Cervantes et al., 2023). The digested samples were compared against calibration standards to determine the total silver content. Vancomycin concentration was determined using a UPLC method adapted from Shou et al. (2014). Furthermore, a fluorescent labeling technique was employed for succinic acid quantification using dansyl chloride (Bartzatt, 2001). The succinic acid concentration in AgNPs was determined to be 0.46 ± 0.06 mg/mL. The concentrations of individual components in the AgNPs/Van, as determined by these analytical methods, are presented in Table 1. This detailed understanding is crucial for optimizing formulation parameters, ensuring batch-to-batch consistency, and correlating composition with biological activity and safety profiles in future studies.

### Structural integrity of AgNP formulations over time and pH variations

**FIGURE 2:**
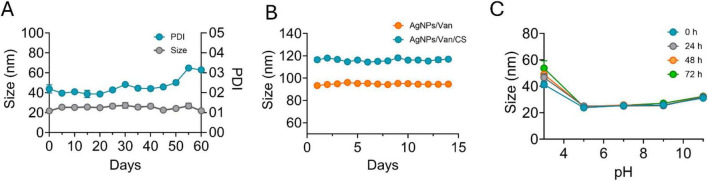
Evaluation of AgNPs stability under various conditions. **(A)** Stability of AgNPs, depicting the particle size and polydispersity index (PDI) of bare AgNPs over a 60-days period when stored at 4 °C in sealed glass vials. **(B)** Stability of functionalized AgNPs over a 14-days period. **(C)** pH stability. All measurements were performed in triplicate, and data are presented as mean ± standard deviation unless otherwise noted.

Maintaining colloidal stability is crucial for preserving the desired properties and biological activities of the nanoparticles. AgNPs are susceptible to aggregation and oxidation, which can markedly influence their characteristics and behavior. There is a notable relationship between AgNPs stability against aggregation and their antimicrobial efficacy. Previous studies have demonstrated that AgNPs aggregation largely affects its antibacterial activity (Bélteky et al., 2019; Levard et al., 2012). In this study, AgNPs stored under refrigerated conditions (4 °C) in sealed, covered glass vials demonstrated stability over 2 months, as evidenced by consistent particle size measurements (Figure 2A). As shown in Figure 2B, AgNPs/Van and AgNPs/Van/CS formulations remained stable for 2 weeks, with only a negligible increase in particle size. To assess the impact of pH on the stability of the AgNPs/Van/CS formulation, addition of either a base (0.1 M NaOH) or an acid (0.1 M HCl) was conducted, and then subjected to particle size analysis at various time points. Our findings revealed that the AgNPs/Van/CS formulation exhibited remarkable stability across a broad pH spectrum (Figure 2C). This pH resilience is a significant characteristic, as it suggests that these nanoparticle formulations may maintain their integrity and functionality in diverse environmental conditions.

### Cytotoxic and hemolytic response to AgNPs-based formulations

**FIGURE 3:**
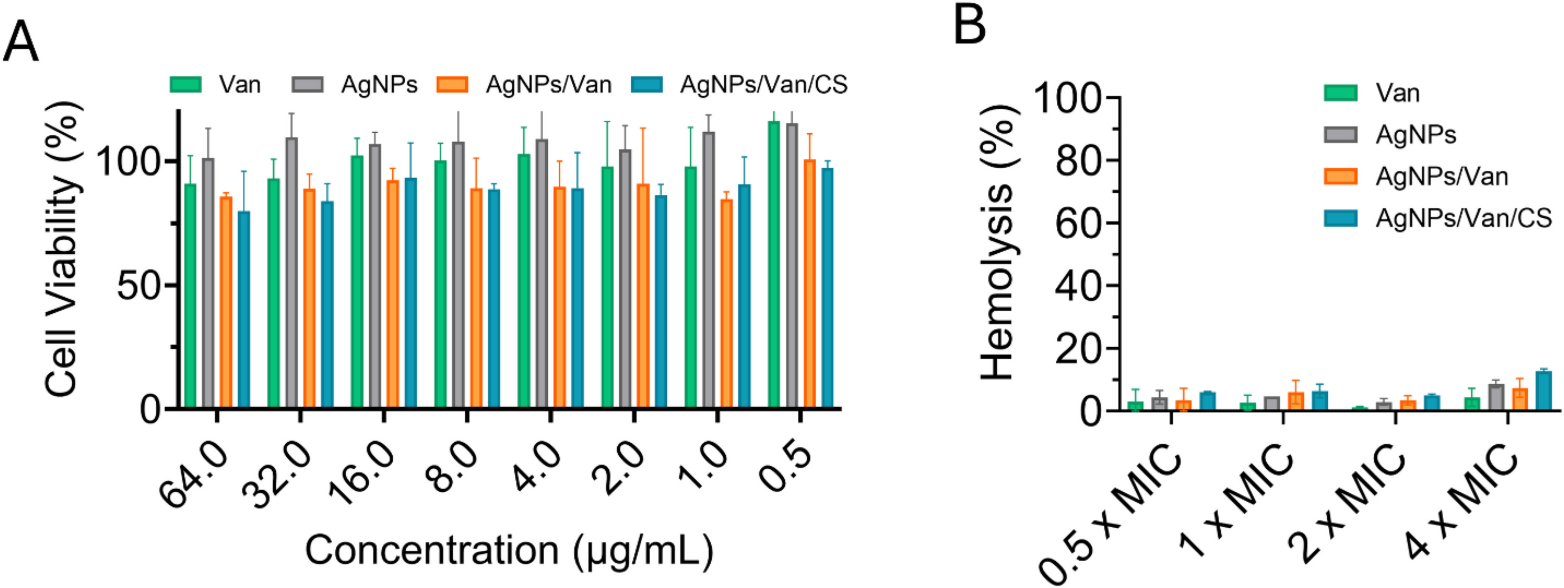
Evaluation of biocompatibility parameters. **(A)** Cell viability. MTT assay conducted on the L-929 cell line over a 24 h period. **(B)** Hemolysis assay. Erythrocyte solutions were incubated for 1 h at 37 °C with either phosphate-buffered saline (PBS) as a negative control or 10% Triton X-100 as a positive control for complete hemolysis. All measurements were performed in triplicate, and data are presented as mean ± standard deviation unless otherwise noted.

Understanding the toxicity profile is imperative for the effective utilization of metallic nanoparticles. To evaluate cell viability at varying vancomycin MIC concentrations, an MTT assay was performed using the murine fibroblast cell line (L-929). Absorbance was measured 24 h after treatment. Results, as illustrated in Figure 3A, revealed that none of the formulations, including those with vancomycin concentrations up to 64 μg/mL, elicit any significant reduction in cell viability. This suggests that within this concentration range, the formulations exhibit a favorable toxicity profile. The hemolytic assay was utilized to evaluate the ability of the formulation to disrupt red blood cell membranes, resulting in the release of hemoglobin into the surrounding medium (Sæbø et al., 2023). Hemoglobin’s characteristic absorption profile enables its quantification through conventional spectrophotometric methods, resulting in optical density (OD) measurements. A solution of 10% Triton X-100 served as the positive control, representing complete erythrocyte lysis (100% hemolysis). Conversely, phosphate-buffered saline (PBS) at physiological pH (∼7) acts as the negative control, indicating the absence of hemolysis (0% lysis). In our study, we observed that the hemolysis ratio for all tested formulations fell within an average range of 5.04% ± 3.21% at different vancomycin MIC concentrations. This finding was derived from the OD 540 nm measurements presented in Figure 3B. Notably, all formulations exhibited similar effects on erythrocyte lysis, as evidenced by the comparable OD values across samples, which suggests a moderate level of interaction with erythrocyte membranes. This data provides valuable insights for determining appropriate dosing strategies in future *in vivo* studies or potential clinical applications.

### Inhibition of *S. aureus* growth by AgNPs/Van/CS

**FIGURE 4:**
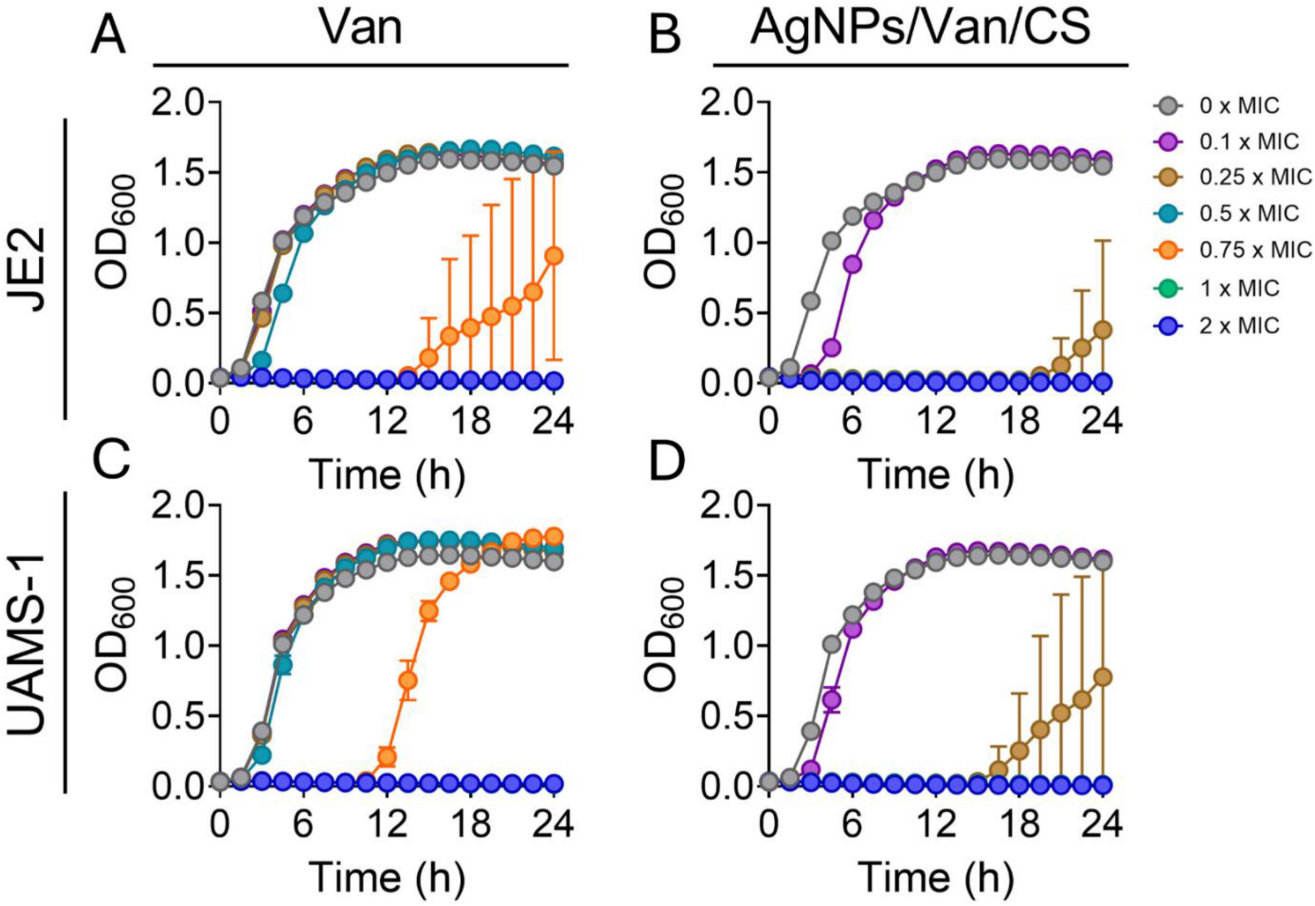
Bacterial growth inhibition by AgNPs/Van/CS. Two *S. aureus* strains, JE2 **(A,B)** and UAMS-1 **(C,D)**, were grown for 24 h in tryptic soy broth, with the addition of vancomycin **(A,C)** or AgNPs/Van/CS **(B,D)** at different vancomycin MIC values. All measurements were performed in quadruplicate, and data are presented as mean ± standard deviation unless otherwise noted.

To evaluate the antibacterial efficacy of AgNPs/Van/CS, we initially analyzed their inhibitory effect on the growth of *S. aureus* strains UAMS-1 (MSSA) and USA300 LAC JE2 (MRSA). Bacterial growth dynamics was monitored over 24 h in liquid cultures exposed to AgNPs/Van/CS and compared to control samples, including untreated cultures and those treated with vancomycin alone (Figure 4). The results of these experiments revealed that AgNPs/Van/CS significantly enhanced bacterial growth inhibition compared to vancomycin alone, demonstrating more than a three-fold improvement in efficacy against both MSSA and MRSA strains (Figure 4). Vancomycin alone exhibited significant growth inhibition at 0.75× MIC, achieving complete inhibition at 1× MIC (Figures 4A, C). In contrast, AgNPs/Van/CS containing vancomycin at just 0.25× MIC demonstrated significant growth inhibition at this concentration and complete inhibition at 0.5× MIC (Figures 4B, D). Notably, this enhanced efficacy was observed regardless of the methicillin resistance profile of the strain. Furthermore, our results indicate that treatment with vancomycin at 0.75× MIC can lead to the emergence of resistance-conferring mutations, whereas with AgNPs/Van/CS such mutations appeared only at a substantially lower concentration of 0.25× MIC. Exposure to vancomycin at 0.75× MIC imposes strong but subinhibitory selective pressure that suppresses fully susceptible cells while allowing less susceptible subpopulations to grow and gradually accumulate, as reflected by the high standard deviation observed at later time points (Figures 4A, B, D; Cui et al., 2003). Under these conditions, *S. aureus* activates cell wall-targeted adaptive responses, including *walKR/vraSR*-associated wall thickening and remodeling, together with *graSR*-mediated surface charge modifications that hinder vancomycin penetration (Cui et al., 2003; Howden et al., 2008; Hu et al., 2016; Wang and Sun, 2021; Yang et al., 2012, 2024). These regulatory shifts enable survival at progressively higher drug concentrations and are reflected in the stepwise MIC increases observed in Figure 4, consistent with the development of adaptive vancomycin resistance at 0.75× MIC.

**FIGURE 5:**
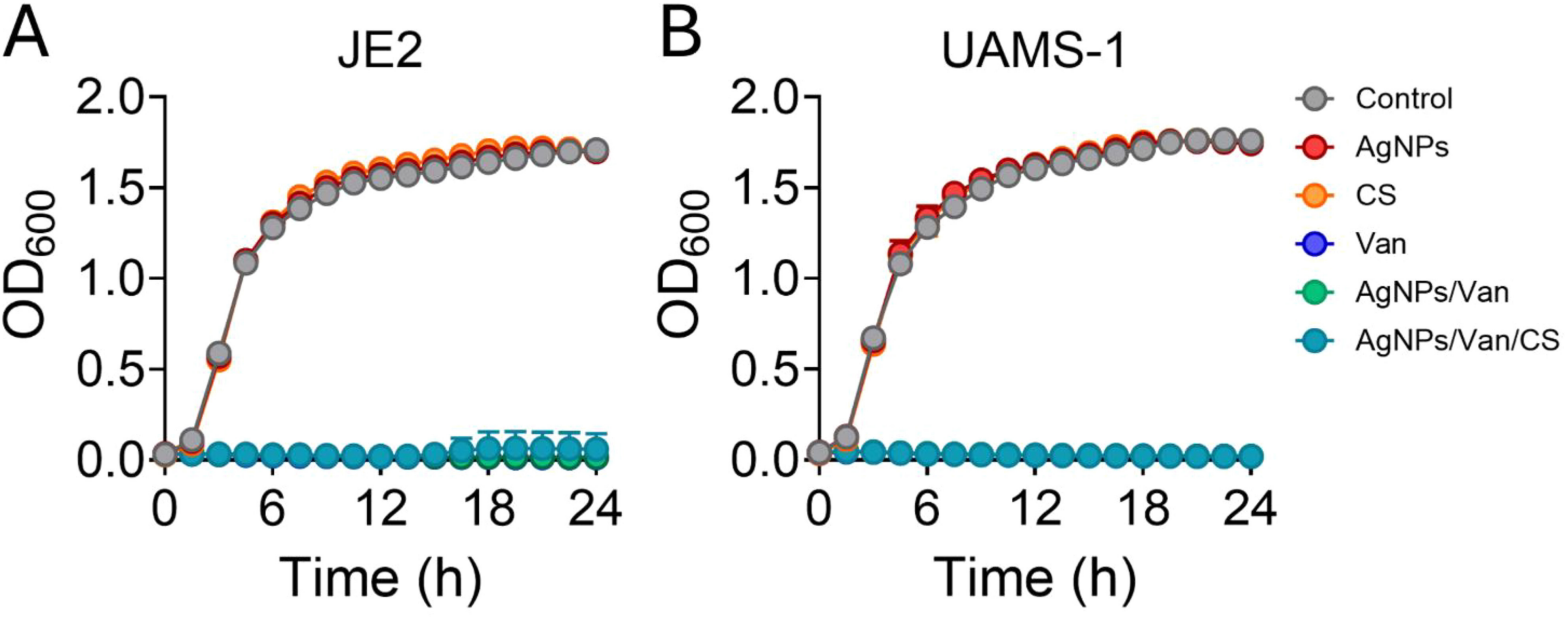
Evaluation of bacterial growth inhibition by individual components used in AgNPs/Van/CS synthesis. *S. aureus* strains JE2 **(A)** and UAMS-1 **(B)** were grown for 24 h in tryptic soy broth supplemented with: AgNPs, chitosan (CS), vancomycin, or AgNPs/Van at the same concentration ratios used in AgNPs/Van/CS, where vancomycin was maintained at 1× MIC. All measurements were performed in quadruplicate, and data are presented as mean ± standard deviation unless otherwise noted.

To further elucidate the mechanism underlying this enhanced activity, we assessed the impact of individual AgNPs/Van/CS components (Figure 5). AgNPs and chitosan alone, at concentrations equivalent to those used in the AgNPs/Van/CS, did not affect bacterial growth, while vancomycin alone, AgNPs/Van, and AgNPs/Van/CS all completely inhibited growth at 1× MIC for vancomycin. Thus, the combination of AgNPs and vancomycin in AgNPs/Van/CS result in greater inhibition than vancomycin alone.

**FIGURE 6:**
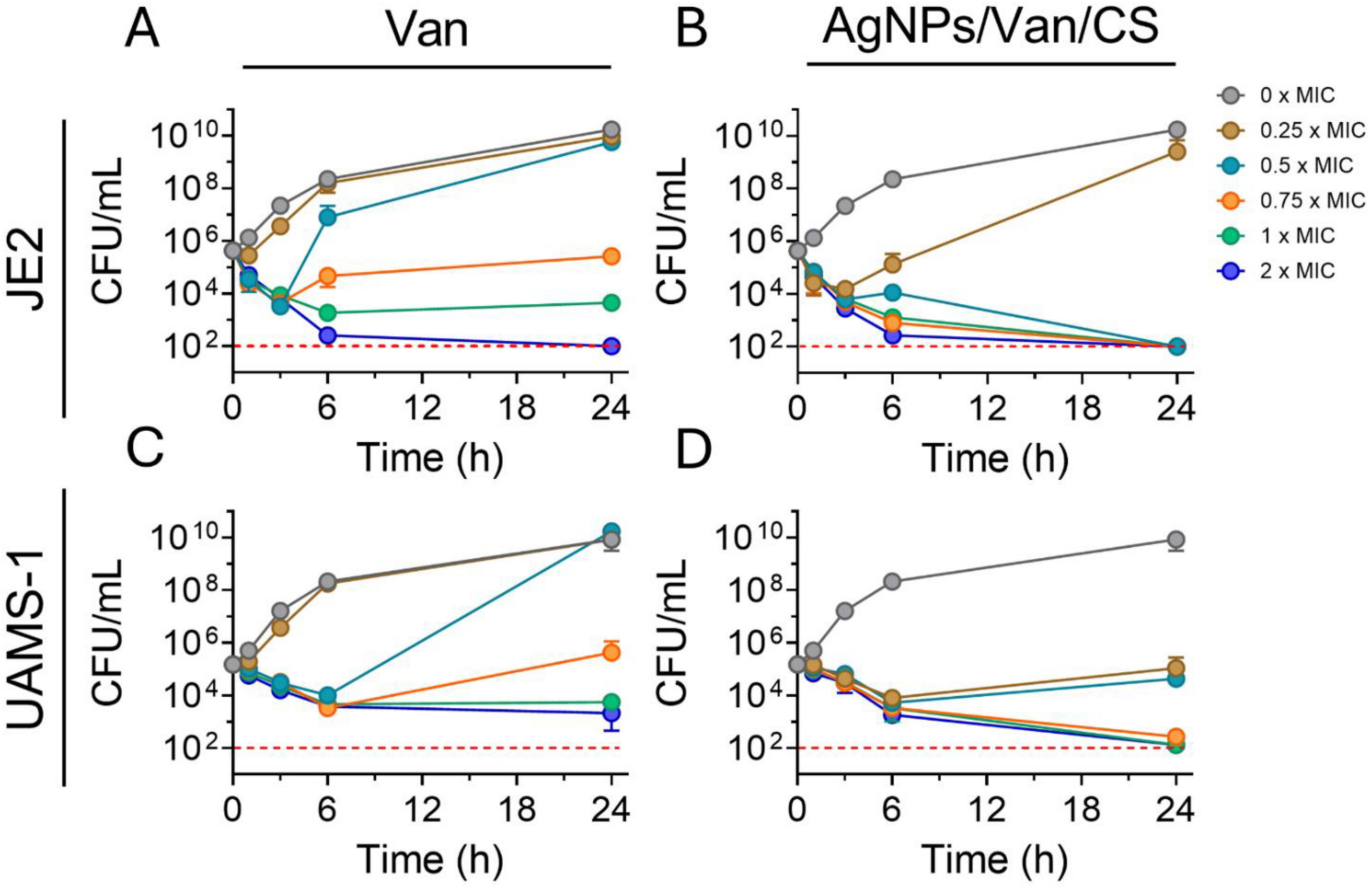
Bactericidal and synergistic effects of AgNPs/Van/CS. *S. aureus* JE2 **(A,B)** and UAMS-1 **(C,D)** were challenged with vancomycin **(A,C)** or AgNPs/Van/CS **(B,D)** for 24 h at the indicated vancomycin MIC values. Colony forming units (CFUs) were determined at 0, 1, 3, 6, and 24 h for each strain and treatment condition. The dashed line indicates the limit of detection at 10^2^ CFU/mL. All measurements were performed in triplicate, and data are presented as mean ± standard deviation unless otherwise noted.

To assess whether the AgNPs/Van/CS combination is synergistic as well as determine its bactericidal potential, we performed time-kill assays using *S. aureus* JE2 and UAMS-1 strains and compared the kinetics of killing to vancomycin alone (Figure 6). As expected, both vancomycin and AgNPs/Van/CS achieved ≥1.5-log_10_ reductions in CFU/mL from the initial inoculum (5 × 10^5^ CFU/mL) at concentration ≥ 1× MIC in both strains by 24 h (Figure 6). However, only the AgNPs/Van/CS combination produced ≥3-log_10_ reduction at sub-MIC concentrations (at 0.5× and 0.75× MIC) in JE2, indicating enhanced lethality of the combination (Figure 6B). A similar trend was observed in UAMS-1, although the bactericidal effect was slightly less pronounced compared to JE2 (Figure 6D).

More importantly, we observed clear synergy of the AgNPs/Van/CS relative to vancomycin alone in time-kill assays of both *S. aureus* strains, defined here as a ≥2-log_10_ reduction in CFU/mL (Belley et al., 2008) at 24 h, with various sub-MIC concentrations of AgNPs/Van/CS (Figures 6B, D). Together, these data demonstrate that the AgNPs/Van/CS combination acts synergistically to enhance killing of both MRSA and MSSA strains.

**FIGURE 7:**
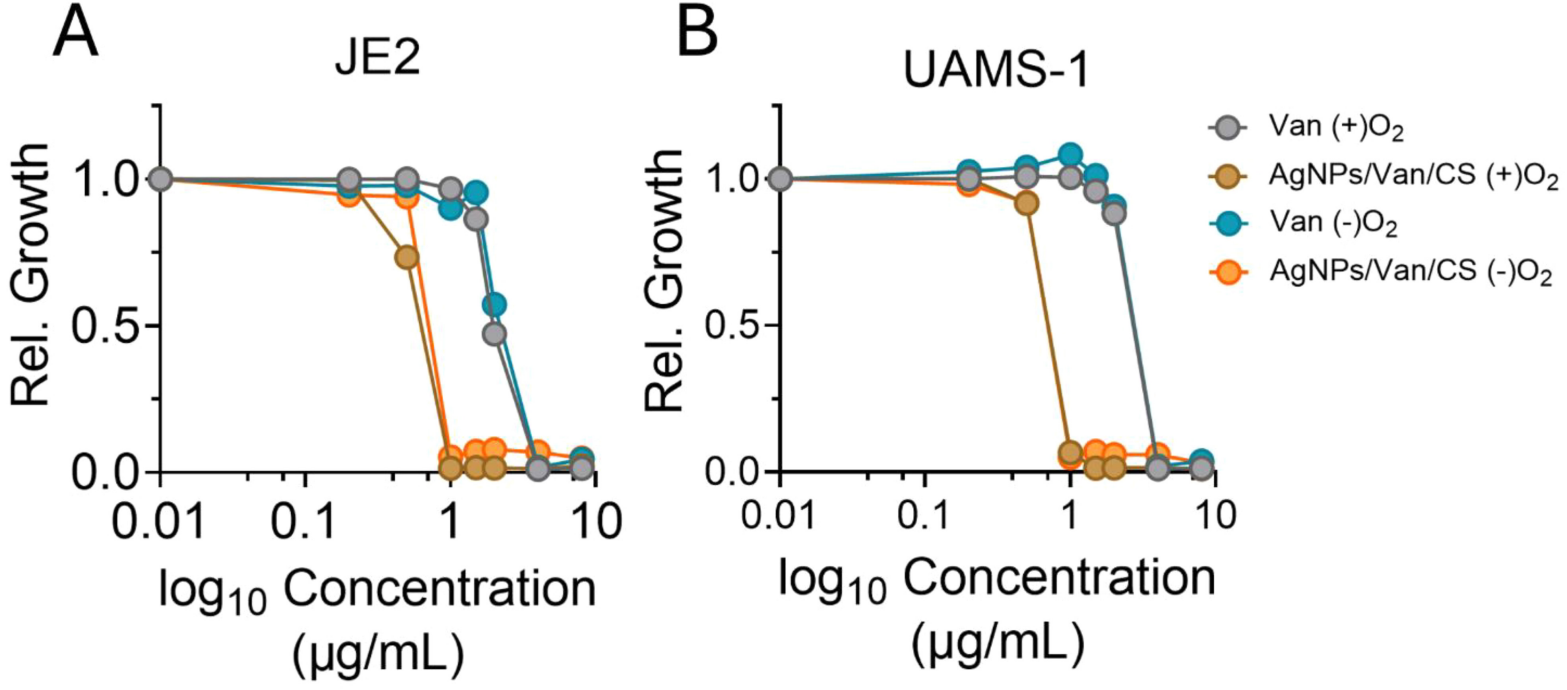
Synergy of AgNPs/Van/CS is independent of ROS production and bacterial respiration. The relative growth of JE2 **(A)** and UAMS-1 **(B)** treated with vancomycin or AgNPs/Van/CS under aerobic and anaerobic conditions was determined from the area under the OD600 growth curve at various antibiotic concentrations, as previously described (Thomas et al., 2013). The relative growth was calculated as the ratio of antibiotic treated to untreated cultures and plotted against antibiotic concentration, enabling normalization for growth differences observed under aerobic and anaerobic conditions. All measurements were performed in triplicate, and data are presented as mean ± standard deviation unless otherwise noted.

The mechanistic basis underlying the enhanced activity of the AgNPs/Van/CS combination relative to vancomycin alone remains unclear. Previous studies have shown that Ag may inhibit respiration or promote reactive oxygen species (ROS) production in bacteria (Bruna et al., 2021; Mazur et al., 2020; Vila Domínguez et al., 2020), potentially contributing to increased lethality when combined with vancomycin. To test these possibilities, we evaluated the growth of JE2, and UAMS-1 strains treated with vancomycin and AgNPs/Van/CS under anaerobic conditions. We reasoned that if ROS generation contributed to the lethality of AgNPs/Van/CS, oxygen limitation would suppress ROS production and restore growth to levels observed with vancomycin alone. Alternatively, if AgNPs/Van/CS inhibits respiration, then vancomycin-treated cells under anaerobic conditions should phenocopy the enhanced growth inhibition observed with the AgNPs/Van/CS combination. However, we found that oxygen limitation did not alter the enhanced growth inhibition of the AgNPs/Van/CS combination compared to vancomycin alone, as observed under aerobic growth in both JE2 and UAMS-1 (Figure 7). These findings indicate that the observed synergy of the AgNPs/Van/CS combination occurs independent of ROS production and respiratory status. Thus, we speculate that enhanced lethality of AgNPs/Van/CS likely arises from the ability of AgNPs to compromise the bacterial cell envelope (More et al., 2023), thereby facilitating increased cell wall penetration of vancomycin against both methicillin-sensitive and methicillin-resistant *S. aureus* than vancomycin alone.

### AgNPs/Van/CS suppress the growth of *S. aureus* clinical isolates

**FIGURE 8:**
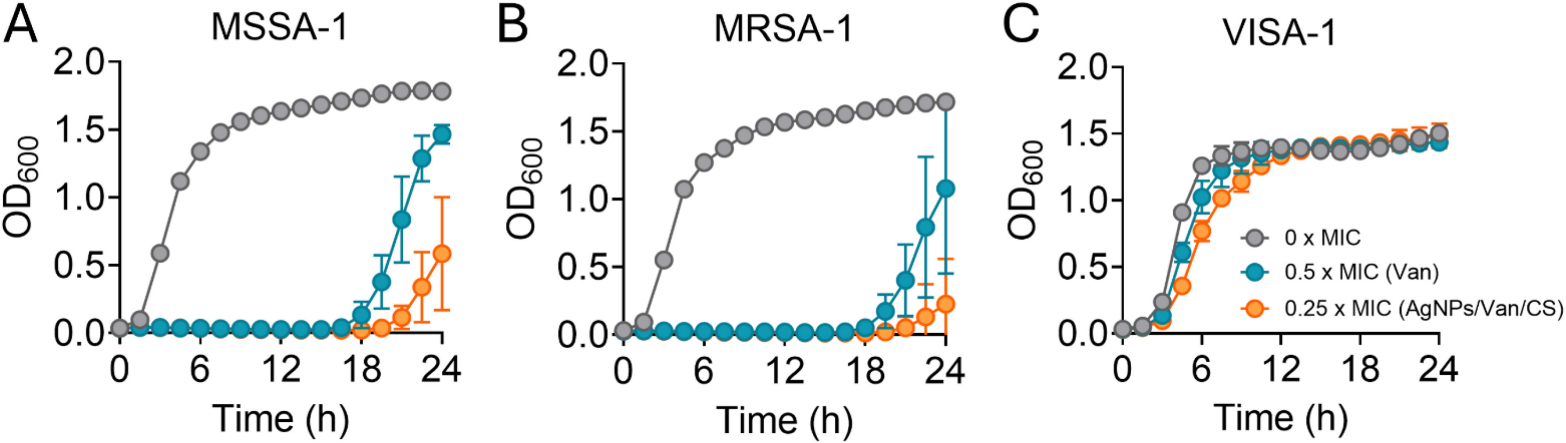
Growth inhibition of *S. aureus* clinical isolates by AgNPs/Van/CS. Clinical isolates MSSA-1 **(A)**, MRSA-1 **(B)**, and VISA-1 **(C)** were grown for 24 h tryptic soy broth supplemented with vancomycin (0.5× MIC) or AgNPs/Van/CS (containing vancomycin at 0.25× MIC). All measurements were performed in quadruplicate, and data are presented as mean ± standard deviation unless otherwise noted.

To evaluate the therapeutic potential of AgNPs/Van/CS against *S. aureus* clinical isolates, we examined its efficacy against MSSA, MRSA, and VISA strains. Our results demonstrated that AgNPs/Van/CS at 0.25× MIC achieved greater growth inhibition of MRSA and MSSA strains than 0.5× MIC of vancomycin (Figures 8A, B), although this effect was less pronounced compared to the results presented in Figure 4. Additionally, AgNPs/Van/CS exhibited a slightly greater inhibitory effect on the VISA strain than vancomycin alone (Figure 8C). These findings suggest that AgNPs/Van/CS maintain strong activity against resistant clinical isolates and show promising efficacy against VISA strains. By enabling effective bacterial suppression at lower vancomycin concentrations, AgNPs/Van/CS have the potential to mitigate resistance development and reduce antibiotic-associated toxicity, highlighting their promise as a strategy for managing challenging antibiotic-resistant *S. aureus* infections.

### Inhibition of *S. aureus* biofilm development by AgNPs/Van/CS

**FIGURE 9:**
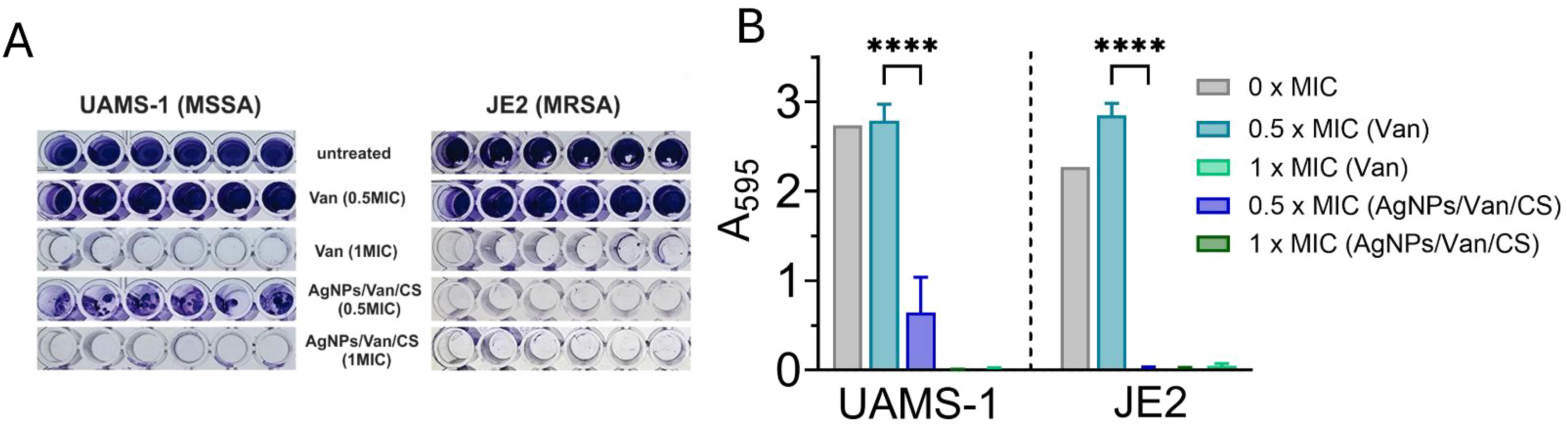
Antibiofilm activity of AgNPs/Van/CS against MSSA and MRSA strains. **(A)** Representative images of static biofilms visualized by crystal violet (CV) staining. The intensity of the staining reflects the extent of biofilm biomass formed by *S. aureus* strains. **(B)** Quantification of biofilm biomass based on CV staining. After staining, absorbance was measured spectrophotometrically to provide a quantitative assessment of biofilm formation. The graph compares the antibiofilm efficacy of AgNPs/Van/CS nanocomposites and vancomycin alone. Data was analyzed by one-way ANOVA with multiple comparisons; *P* < 0.0001. All measurements were performed in triplicate, and data are presented as mean ± standard deviation unless otherwise noted. ****P < 0.0001.

To evaluate the efficacy of AgNPs/Van/CS in inhibiting biofilm formation by MSSA and MRSA strains, we conducted static biofilm assays and quantified biofilm biomass after 24 h using crystal violet staining (Figure 9A). Results showed that both vancomycin and AgNPs/Van/CS at 1× MIC completely prevented biofilm formation in both strains (Figures 9A, B). Notably, at a subinhibitory concentration of 0.5× MIC of vancomycin, AgNPs/Van/CS significantly inhibited biofilm development by MSSA (UAMS-1), reducing the final biofilm biomass by approximately three- to six-fold, while at the same concentration it completely inhibited biofilm formation by the MRSA (JE2) strain (Figure 9B). In contrast, vancomycin alone at 0.5× MIC had no discernible effect on biofilm development in either strain (Figures 9A, B). The observed differences in the antibiofilm efficacy of AgNPs/Van/CS at 0.5× MIC between UAMS-1 and JE2 may be attributed to strain-specific variations in biofilm matrix composition, such as differences in surface protein expression, polysaccharide intercellular adhesin (PIA) production, or other extracellular matrix components (Cue et al., 2012; McCarthy et al., 2015; Moormeier and Bayles, 2017; O’Gara, 2007). Specifically, MSSA strains like UAMS-1 primarily rely on PIA for biofilm stability, whereas MRSA strains such as JE2 depend more on surface proteins (e.g., SasG, Bap) and extracellular DNA (eDNA) for matrix integrity (Hernández-Cuellar et al., 2023). These findings demonstrate that AgNPs/Van/CS exhibit enhanced antibiofilm activity at sub-MIC levels compared to vancomycin alone and suggest that AgNPs/Van/CS may disrupt biofilm architecture development or interfere with early adhesion processes. This positions AgNPs/Van/CS as a promising strategy for combating biofilm-associated *S. aureus* infections, particularly in settings where conventional antibiotics fail to prevent biofilm formation or eradicate mature biofilms.

## Conclusion

This study underscores the potential of AgNPs/Van/CS as a highly effective antimicrobial strategy, enabling bacterial suppression at lower vancomycin MICs. This approach represents an advancement in the ongoing battle against antibiotic-resistant bacteria, leveraging the unique antimicrobial properties of metallic nanoparticles in conjunction with conventional antibiotic therapy. The findings also support the potential of nanoparticle-based delivery to improve antimicrobial efficacy and represent a proof-of-concept for this combination strategy. While the results are promising, the current study is limited to *in vitro* evaluation. Key aspects required for clinical translation including *in vivo* efficacy, pharmacokinetics/pharmacodynamics (PK/PD), immunotoxicity, long-term safety, and resistance evolution have not yet been assessed and warrant further investigation. Overall, this work provides a foundation for future studies aimed at evaluating the therapeutic potential of AgNPs/Van/CS in more complex biological system.

## Data Availability

The raw data supporting the conclusions of this article will be made available by the authors, without undue reservation.
